# Supplementary material for: Combining information from parental and personal experiences: Simple processes generate diverse outcomes
Source: PLoS One. 2021 Jul 13;16(7):e0250540. doi: 10.1371/journal.pone.0250540 (PMC8277055; doi:10.1371/journal.pone.0250540)
Supplement: S1 Appendix — (DOCX) [file pone.0250540.s001.docx]

**S1 Appendix: List of terms**

**Inductive experience**: An experience which induces changes in the development of phenotypic traits in an organism. When an inductive experience provides information about the value of a state of the external environment, it is called a ‘cue’.

**Likelihood** (likelihood function): a distribution that describes the conditional probability that a particular experience (e.g., exposure to a particular concentration of kairomones from a predator for a particular period of time) will occur, given each of the 100 possible values of a state of the environment (e.g., predator density). In this article, the shape of each likelihood function is modelled using a beta distribution, which is described by two parameters: α and β. Here, a ‘cumulative’ likelihood function describes the conditional probability that an individual would have all of the experiences to which it is exposed in a P or in an N treatment, given each of the possible values of the state. See the S2 Figure for illustrations of the likelihood functions used in this article.

**N- models**: Models in which the information provided by the conditions in the N treatment is much less reliable than the information provided by the conditions in the P treatment

**N* models**: Models in which the information provided by the conditions in the N treatment and the information provided by the conditions in the P treatment are equally reliable

**NN**: An experimental treatment in which neither parents nor offspring are exposed to cues from predators

**NP**: An experimental treatment in which parents are not exposed to cues from predators, offspring are

exposed to those cues

**Naïve Prior:** A distribution that indicates an individual’s assessment of the probability of each of the possible values of a state before that individual has been personally exposed to any informative experiences. The naïve prior can be based on information provided by genes, inherited epigenetic factors, parental experiences, and other factors.

**Offspring Estimate**: Here, we use ‘offspring estimate’ to indicate the mean of the offspring’s posterior distribution at the end of the experiment.

**Prior**: Here, we use Prior to indicate the parental Prior: a distribution which indicates the parent’s initial assessment of the probability of each of the possible values of a state of the environment (e.g., predator density) at the beginning of an experimental study of TWP. This prior is based on the parent’s naïve prior, as well as on any informative experiences that occurred to the parent before the beginning of the experiment. The mean of a parental prior indicates the parent’s best point estimate of the value of the state; the variance of a parental prior indicates the parent’s level of confidence in that estimate. Because we set the variance at the same value (0.04) for all of the Priors, in the text and figures we focus on Priors with different means (0.1, 0.5, and 0.9). See the S1 Figure for illustrations of these Priors.

**Posterior distribution**: a distribution which indicates an individual’s assessment of the probability of each of the possible values of a state after that individual has been exposed to an informative experience

**PN**: An experimental treatment in which parents are exposed to cues from predators, offspring are not

**PP**: An experimental treatment in which both parents and offspring are exposed to cues from predators

**State of the environment**: A variable in the external environment (e.g., predator density). We assume that this variable can take on one of many possible values (for instance, predator density can range from 0 to some maximum possible value).

**TGP**: Trans-generational plasticity

**TWP**: Trans and within-generational plasticity

**WGP**: Within generational plasticity
