# Supplementary material for: Combining information from parental and personal experiences: Simple processes generate diverse outcomes
Source: PLoS One. 2021 Jul 13;16(7):e0250540. doi: 10.1371/journal.pone.0250540 (PMC8277055; doi:10.1371/journal.pone.0250540)
Supplement: S2 Appendix — (DOCX) [file pone.0250540.s002.docx]

**S2 Appendix: Estimating posterior probabilities using Bayesian estimation**

Our analysis is based on the assumption that individuals estimate the value of a state *A* (e.g., predator density), and that these values can vary continuously between a minimum and a maximum. However, for the purposes of computing the posterior probability, it is easier to discretize the variable into a finite number of small, mutually exclusive values of the state. We assume that the minimum and maximum values of the state are *A_min_*=0 and *A_max_* = 1. Thus, when we discretize the variable into *n* = 100 equally-spaced values between its minimum value and maximum value, the *i*-th state will be bounded by (0.01(*i*-1), 0.01*i*). If *A_1_, A_2_,……A_n_* are the *n* individual values, then the probability that the value of the state is *A_i_* is described by *P*(*A_i_*). Also, since *A_1_, A_2_,……A_n_* represent all of the possible values of the variable, the sum of their probabilities will add up to 1, i.e.,

$P\left( A_{1} \right)+P\left( A_{2} \right)+\ldots+P\left( A_{n} \right)=1$

P(B) is the probability that experience B will occur. The probability of B given variable A, is given by the Law of Total Probabilities as $\left( B/A \right)=P\left( A_{1} \right)P\left( B/A_{1} \right)+P(A_{2})P\left( B/{A_{2}} \right)+\ldots+P(A_{n})P(B/A_{n})$ , where *P*(*B/A_i_*) represents the conditional probability of *B* given the value *A_i_*. The conditional probability function relating the experience to the variable, i.e., *P* (*B/A*), is called the likelihood function. Using the Bayes’ theorem, we can now estimate the posterior probability of any given value of the state, *A_i_* given the occurrence of the experience, *B*, as $P\left( {A_{i}}/B \right)= \frac{P\left( A_{i} \right)P(B/{A_{i}})}{P(B)}$

In our analysis, we use the beta distribution to describe the shapes of both the initial prior distribution (here, the parental prior) and the cumulative likelihood functions for the experiences to which parents or offspring were exposed during the rearing period. The general form of the probability density function for a beta distribution is with 0 ≤ *x* ≤ 1 and $p\left( x \right)$ = 0 for all *x* outside this range, where *Γ* represents the Gamma function, and *α* and *β* are parameters of the beta distribution. Because the current analysis involves two different beta distributions, we describe the shape of the parent’s prior distribution using a beta distribution in which *α = a* and *β = b* and the shape of the cumulative likelihood function using a second beta distribution in which *α = c* and *β= d*.

For *n* = 100, for the prior distribution we can numerically approximate the probability *P*(*A_i_*) by where *p_A_*(*x*) is the probability density function for the first beta distribution. Similarly, the probability of occurrence of the experience, *B*, given the *i*-th state, *A_i_*, can be computed as , where *p_B_*(*x*) is the probability density function for the second beta distribution.

The posterior probabilities of each of the states can then be computed as follows. First, we start with the parental prior distribution. If we indicate the midpoint of the *i*-th value as $y_{i}=(0.01i-0.005)$, and *A_1_, A_2_,...,A_n_* are the *n* values associated with variable *A*, then the prior probability distribution may be computed using the standard beta probability density function as

Similarly the conditional probability, *P*(*B/A_i_*), for each value can be computed using the beta probability function with parameters *c* and *d* as

Based on Bayes’ theorem, the posterior probability of each value is given by

$P\left( {A_{i}}/B \right)= \frac{P\left( A_{i} \right)P(B/{A_{i}})}{P(B\boldsymbol{)}}$ , for *i* = 1,2,…,*n*

Finally, we need to normalize these posterior probabilities, so that the sum $P\left( A_{1} \right)+P\left( A_{2} \right)+\ldots+P\left( A_{n} \right)=1$. Normalization is required because we have specified that *A_1_, A_2_, …., A_n_* cover all of the possible values of the variable *A*, so that the sum of the probabilities of these values must add up to 1 for any prior or posterior distribution. We normalize the posterior probabilities by dividing each *P* (*A_i_*/*B*) by the term

$\sum_{i=1}^{100} P(A_{i}/B)$ , so that the final posterior probabilities are given by

$$\frac{P({A_{i}}/B)}{\sum_{i=1}^{100} P({A_{i}}/B)}= \frac{P\left( A_{i} \right)P(B/{A_{i}})}{P(B)}\frac{P(B)}{\sum_{i=1}^{100} P\left( A_{i} \right)P(B/{A_{i})}}= \frac{P\left( A_{i} \right)P(B/{A_{i}})}{\sum_{i=1}^{100} P\left( A_{i} \right)P(B/{A_{i})}}$$

The posterior distributions generated by this procedure are unlikely to be beta distributions, even though the initial prior distribution was a beta distribution. However, we can solve numerically for the posterior distributions that occur as a function of experiences of parents and offspring, because each successive posterior distribution is available as a computed distribution at the end of the process described above.

Notes

1. Although *P*(*B*) can take on many different values, the value of *P*(*B*) does not affect the computation of the posterior distribution, because of the process of normalizing the posterior probabilities (see equation above).

2. An alternative method for computing the distributions for *P*(*A_i_*) and *P*(*B*/*A_i_*) would be to use the cumulative probability distribution function for a beta distribution. For example, the prior distribution can be computed as *P*(*A_i_*) = betaCDF(*0.01i,α,β*) – betaCDF(*0.01(i-1),α,β*), where betaCDF is the beta cumulative distribution function with parameters α and β. This function is available in most standard statistical packages (e.g. R, SAS).
