# Supplementary material for: Combining information from parental and personal experiences: Simple processes generate diverse outcomes
Source: PLoS One. 2021 Jul 13;16(7):e0250540. doi: 10.1371/journal.pone.0250540 (PMC8277055; doi:10.1371/journal.pone.0250540)
Supplement: S3 Appendix — (DOCX) [file pone.0250540.s003.docx]

**S3 Appendix:** **Specifying the cumulative likelihood functions used to describe the information provided by the parent’s and offspring’s experiences in studies of TWP**

In our models of TWP, the ‘experience’ (indicated by *B* in the S2 Appendix) consists of all of the conditions to which the parents or the offspring were exposed during their respective P or N treatments. In this situation, the ‘cumulative likelihood function’ for a given treatment (e.g., for the P treatment) describes the conditional probability *P*(*B/A_i_*) that subjects would have all of the experiences to which they were exposed in that treatment (e.g., constant exposure to a high concentration of kairomones from a predator for four months), given each of the 100 possible values of the state (*A_i_*). In the baseline models, we assumed that parents and offspring were exposed to identical conditions for the same period of time in their respective P and N treatments, so we used the same beta distributions to describe the shapes of the cumulative likelihood functions for the P treatment and for the N treatment for both generations (see Stamps and Bell, 2020).

In order to model experiments in which parents or offspring were exposed to the same cues for different periods of time, we divided each treatment period into four intervals of equal length. In this case, the experience *B* either consisted of exposure to the cues for one interval, or no exposure to the cues for one interval. We first specified the likelihood function for one interval with cues and for one interval without cues. For instance, for an interval with cues, we specified a beta distribution which described the conditional probability *P*(*B/A_i_*) for each value of the state that the subjects would be exposed to the cues for one interval. Then, for each generation, we determined the beta distribution which described the cumulative likelihood function for the conditional probability that the subjects would have the set of experiences specified in the text (e.g., exposure to the cues for two intervals, and no exposure to the cues for two intervals), given each value of the state.

In order to model situations in which the information from parents was devalued or degraded, we assumed that the cumulative likelihood function for the parents’ experiences in their P treatment and the cumulative likelihood function for the offsprings’ experiences in their P treatment had the same mean, but that the variance of the cumulative likelihood function based on the parents’ experience was higher than the variance of the cumulative likelihood function based on the offsprings’ experience. We did this by first selecting a beta distribution to describe the shape of the cumulative likelihood function for the offspring, and then finding the beta distribution that had the same mean value, but a higher variance than the beta distribution that was used to describe the shape of the cumulative likelihood function for the offspring. See the S2 Figure for examples of cumulative likelihood functions with the same mean and different variances.
