# Supplementary material for: Combining information from parental and personal experiences: Simple processes generate diverse outcomes
Source: PLoS One. 2021 Jul 13;16(7):e0250540. doi: 10.1371/journal.pone.0250540 (PMC8277055; doi:10.1371/journal.pone.0250540)
Supplement: S4 Appendix — (DOCX) [file pone.0250540.s004.docx]

S4 Appendix: Pseudo-code for the Matlab program used to analyze TWP

We assumed that there are 100 discrete values of a state of the environment (e.g., predator density). For each ‘animal’, the user specifies an initial parental prior distribution (Prior); this distribution indicates the parent’s initial estimate of the probability that each of the values of the state is the ‘true’ value of the state at the current locality. We assumed that the shape of parental Priors conforms to a shape which can be specified by a beta distribution. The shapes of the parental Priors used in the current study are illustrated in the S1 Figure.

We assumed that the subjects in each generation had a set of experiences in their respective treatments. For each of the 100 possible values of the state, we computed the conditional probability that those experiences would occur, given that each of those values was the true value of the state. We assumed across all of the 100 possible values of the state, the conditional probabilities of the experiences given each value conformed to a shape specified by a beta distribution. This is the cumulative likelihood function for a given set of experiments. Examples of the shapes of the cumulative likelihood functions used in the current study are illustrated in the S2 Figure.

In this program, the user specifies the number of animals, and the mean and the variance of the initial prior distributions for each animal; these are converted within the program to the beta distributions with those means and variances. The user enters the alpha and the beta parameters for the beta distributions used to specify the shapes of the cumulative likelihood functions for the four treatments in factorial studies of TWP: parents exposed to cues, parents not exposed to cues, offspring exposed to cues and offspring not exposed to cues. Here, A indicates one rearing condition (e.g., reared with cues); B indicates the other rearing condition (e.g., reared without cues), 1 indicates the parent's experiences, and 2 indicates the offspring experiences. For the cumulative likelihood functions for each of the four treatments, the user specifies the two parameters that are required to specify the shape of any beta distribution. For each likelihood function, we use "c" to indicate the value of the alpha parameter for the beta distribution, and "d" to indicate the value of the beta parameter for the beta distribution.

The program plots the means of the prior or the posterior distributions for the four combinations of parental and offspring experiences (B1,B2; A1,B2; A2,B1; A1,B1), at three different points in time

(age 0 before the parents have been exposed to the experiences), age 1 (after the parent's exposure to the experiences) and age 2 at the end of the offspring experiences).

See Appendix S5 for additional information.

* Italics indicate abbreviations for the names of variables

* {} indicates start and end of a loop.

Define Inputs *c* & *d* for *A1*, *A2*, *B1* and *B2*

Define *maxage* to be 2

{FOR LOOP LAYER 1 – For each number of combinations

Case 1 *c* = [*B1c B2c*]; *d* = [*B1d b2d*];

Case 2 *c* = [*A1c B2c*]; *d* = [*A1d B2d*];

Case 3 *c* = [*B1c a2c*]; *d* = [*B1d A2d*];

Case 4 *c* = [*A1c A2c*]; *d* = [*A1d A2d*];

Define the number of animals to be 3.

Define the number of values of the state of the environment to be 100.

Define the list of the mean values for the priors for each animal to be [0.1, 0.5, 0.9].

Define the list of the variances of the priors for each animal to be [0.04, 0.04, 0.04].

{FOR LOOP LAYER 2: for *i* = each value of the state

$x\left( i \right)=\frac{1}{value of states}*i$

$midx\left( i \right)=x\left( i \right)-\frac{1}{2*value of states}$

ENND OF FOR LOOP LAYER 2}

{For loop layer 2: For *janm* = 1 to max number of animals

Set *actage* = 0

Set *ageplus* = 1

Read mean and variance set above ([0.1, 0.5, 0.9], [0.04, 0.04, 0.04])

$a=\frac{\left( 1-mean \right)*{mean}^{2}-mean*variance}{variiance}$

$b=\frac{\left( 1-mean \right)*a}{variiance}$

Calculate *prior*(1) as the beta CDF at *x*(1) under the *a* as α and *b* as β calculated under the current loop.

And prior (2 to value of state) as beta CDF at *x*(i) – beta CDF at *x*(i-1) under the *a* as α and *b* as β calculated under the current loop.

Get animal prior mean: $mean(janm)= midx*piror'$

{FOR LOOP LAYER 3: For i = 1 to max value of states

$varl\left( i \right)= varl\left( i-1 \right)+midx\left( i \right)-{mean(janm)}^{2}*prior(i)$ where $varl\left( 1 \right)=0$

END OF FOR LOOP LAYER3}

Get animal prior variance: $var\left( janm \right)=varl(last looped one)$

{FOR LOOP LAYER 3: For age = 1 to max age

Set $ageplus=age+1$

Calculate *posterior*(1) as the beta CDF at *x*(1) under the *c(age)* as α and *d(age)* as β which defined at beginning cases.

And *prior* (2 to max value of state) as beta CDF at *x*(i) – beta CDF at *x*(i-1) under the *c*(age) as α and *d*(age) as β which defined at beginning cases.

{FOR LOOP LAYER 4: For i = 1 to max value of the state

$post\left( i \right)=prior\left( i \right)*posterior(i)$

END OF LOOP LAYER 4}

Normalize the posterior probability, sum the posterior probability $sumpost=sum all post\left( i \right)$

{FOR LOOP LAYER 4: For i = 1 to max value of the state

$post\left( i \right)=\frac{post\left( i \right)}{sumpost}$

END OF LOOP LAYER 4}

Get animal post mean: $post mean= x*post'$

{FOR LOOP LAYER 4: For i = 1 to max value of states

$post varl\left( i \right)= post varl\left( i-1 \right)+{[x\left( i \right)-post mean]}^{2}*post(i)$

where $var\left( 1 \right)=0$

END OF FOR LOOP LAYER4}

Get animal post variance: $var\left( janm \right)=post varl(last looped one)$

Rest prior to be equal to post calculated.

END OF LOOP LAYER 3}

END OF LOOP LAYER 2}

END OF LOOP LAYER 1}
