# Supplementary material for: Combining information from parental and personal experiences: Simple processes generate diverse outcomes
Source: PLoS One. 2021 Jul 13;16(7):e0250540. doi: 10.1371/journal.pone.0250540 (PMC8277055; doi:10.1371/journal.pone.0250540)
Supplement: S5 Appendix — (DOCX) [file pone.0250540.s005.docx]

S5 Appendix: Matlab code for the program used to compute and plot results for Bayesian analyses of TWP

%Twostage6 Updated 5/18/21

clc;clear all;close all;

% A indicates one rearing condition (here, reared with cues); B indicates

% the other rearing condition (here, reared without cues). 1 indicates the

% parent's experiences, 2 indicates the offspring experiences.

% For instance, B1B2 would indicate that both parents and offspring were

% reared in the absence of cues, A1B2 that parents were reared with cues,

% offspring were reared without cues, and so on.

% The program uses the beta distribution to describe the shape of the

% parent's initial prior distribution. The model assumes that there are 100

% possible mutually exclusive values of a state of the environment

% (e.g. predator density); these values range from 0 to 1. The model

% model asks the user to input desired the mean (mupr1st)and the variance

% (varpr1st) of the parental prior for each ‘animal’ (i.e. for each parent)

% For convenience, we focus on three parental priors, with means of 0.1. 0.5 % and 0.9, and the same variance (.04).

% Then the program computes, for each animal, the alpha and the beta

% parameter values for the beta distribution which

% produces a distribution with the desired mean and variance for each

% animal’s prior

% The program also uses the beta distribution to describe the shape of the

% cumulative likelihood function for the conditions in which the parents or

% the offspring were reared, as specified in the text. Again, we assume that

% there are 100 possible mutually exclusive values of

% the state. The likelihood function indicates the probability that the

% individual will have a given experience (e.g. either be exposed to the cues

% or not be exposed to the cues for specified periods of time), given that

% the true value of the state is each of the 100 possible values.

% For each likelihood function, the user specifies the two parameters

% that are required to specify the shape of any beta distribution.

% For each likelihood function, we use "c" to indicate the value of the alpha

% parameter for the beta distribution, and "d" to indicate the value of the

% beta parameter for the beta distribution. We allow the

% likelihood functions for the parent and offspring generations to differ...

% this allows us to model situations in which the duration of exposure to

% the cues, or the reliability of the information provided by the cues,

% differs for parents and offspring.

% The user inputs 8 values, which indicate the alpha(c)and the beta(d) values

% for the beta distributions for the likelihood functions when the parental

% generation is exposed to cues(A1c and A1d), when the offspring generation

% is exposed to cues (A2c and A2d),when the parental generation is not % exposed to cues (B1c and B1d) and when the offspring generation is not % exposed to cues (B2c and B2d).

% This program plots the means of the prior or the posterior distributions

% for the four combinations of parental and offspring experiences

% (B1B2; A1,B2; A2,B1; A1,B1), at three different points in time

% (age 0 before the parents have been exposed to the experiences),

% age 1 (after the parent's exposure to the experiences) and age 2

% at the end of the offspring experiences). Each 'animal' starts with a

% different parental Prior (here there are 3 animals, with parental priors

% with shapes with means of .1, .5 and .9, and the same variance (0.04).

% We use beta distribution for the likelihood fn. The program uses

% the cumulative Beta distribution, so we need access to betacdf function

%% Input area

A1c=input('Input c for A1:');

A1d=input('Input d for A1:');

A2c=input('Input c for A2:');

A2d=input('Input d for A2:');

B1c=input('Input c for B1:');

B1d=input('Input d for B1:');

B2c=input('Input c for B2:');

B2d=input('Input d for B2:');

A1_value=[A1c A1d];

A2_value=[A2c A2d];

B1_value=[B1c B1d];

B2_value=[B2c B2d];

check_message_A1 = ['A1 input:',mat2str(A1_value)];

check_message_A2 = ['A2 input:',mat2str(A2_value)];

check_message_B1 = ['B1 input:',mat2str(B1_value)];

check_message_B2 = ['B2 input:',mat2str(B2_value)];

disp('Below are your inputs')

disp(check_message_A1);

disp(check_message_A2);

disp(check_message_B1);

disp(check_message_B2);

maxage = 2;

for num_of_combination = 1:4

switch num_of_combination

case 1

c=[B1c B2c];

d=[B1d B2d];

case 2

c=[A1c B2c];

d=[A1d B2d];

case 3

c=[B1c A2c];

d=[B1d A2d];

case 4

c=[A1c A2c];

d=[A1d A2d];

end

nanim = 3; n = 100; **nanim** = number of animals

muprlst=[0.1 0.5 0.9 ]; **n** = number of values of the state

varprlst=[.04 .04 .04 ]; **mupr1st** = the mean of the prior

% Preassign vector sizes for speed for each animal; **varpr1st** = the

x=zeros(1,n); variance of the prior for each

midx=zeros(1,n); animal

prior=zeros(1,n);

pes=zeros(1,n);

post=zeros(1,n);

for i=1:n

x(i)=(1/n)*i;

midx(i)=x(i)-(1/(2*n));

end

xxplot=x;

% %nanim = input('No. of animals = ');

% %maxage = input('Maximum Age of animals = ');

dage = maxage+1;

% preallocating matrix sizes for speed

mean = zeros(nanim,dage);

var=zeros(nanim,dage);

% Start of animal loop

for janm = 1:nanim

actage=0;

ageplus = 1;

% read mupr and varpr from list

mupr = muprlst(janm);

varpr = varprlst(janm);

a = ((1-mupr)*mupr^2 - mupr*varpr)/varpr; Computing the α and

b = ((1-mupr)*a)/mupr; β values for the beta

distribution which produces a parental Prior distribution with the mean and the variance specified by the user

%% New for plot

Data1(janm).animal(num_of_combination).a = a;

Data1(janm).animal(num_of_combination).b = b;

% % disp('animal a b'); % Display parameters for animal

out1= [janm,a,b];

% % disp(out1) Using the beta distribution

prior(1)=betacdf(x(1),a,b); to set up the prior

for each value of the

state,i

for i=2:n

prior(i)= betacdf(x(i),a,b)- betacdf(x(i-1),a,b);

end

% Set up table data

mean(janm,ageplus)= midx*prior';

vartmp=0;

for i=1:n

vartmp = vartmp+(midx(i)-mean(janm,ageplus))^2*prior(i);

end

var(janm,ageplus) = vartmp;

%Display prior data for animal

prdat = [actage mean(janm,1) var(janm,1)];

%% New for plot

Data2(ageplus).age(janm).animal(num_of_combination).mean=mean(janm,1);

Data2(ageplus).age(janm).animal(num_of_combination).var=var(janm,1);

% % disp(' age Mean Variance')

% % disp(prdat);

% Start of age loop

for age= 1:maxage

ageplus = age+1;

actage=age;

% likelihood function for

% %c= input(' Parameter c of likelihood fn = '); the experience in a given

% %d=input('Parameter d of likelihood fn = '); treatment (P or N, for

Parent or Offspring)

pes(1)=betacdf(x(1),c(age),d(age));

for i=2:n

pes(i)= betacdf(x(i),c(age),d(age))- betacdf(x(i-1),c(age),d(age));

end

% compute the posterior prob

for i=1:n for each value of the state,i

post(i) = (prior(i)*pes(i));

end

% Normalize the posterior probabilities

sumpost = sum(post);

for i=1:n

post(i) = post(i)/sumpost;

end

% Plot of functions

% % plot(xxplot, prior,'b',xxplot,pes,'r', xxplot, post, 'g'); pause

% Compute mean and variance of posterior

postmean = x*post';

postvar=0; % initialize the variance variable

for i=1:n

postvar= postvar + ((x(i)-postmean)^2)*post(i);

end

% Display Data

prdat = [actage postmean postvar];

% % disp(prdat);

% Data Table

mean(janm,ageplus) = postmean;

var(janm,ageplus) = postvar;

%% New for plot

Data2(age+1).age(janm).animal(num_of_combination).mean=postmean;

Data2(age+1).age(janm).animal(num_of_combination).var=postvar;

% Reset prior

prior=post;

end % end of age loop

end % end of animal loop

% % disp('c');disp(c); disp('d');disp(d); pause

% % disp('Mean data'); disp(mean); pause

% % disp('Variance Data'); disp(var)

end

for animals = 1:nanim

coef = {'c';'d'};

A1=A1_value';

A2=A2_value';

B1=B1_value';

B2=B2_value';

eval(['Animal_',mat2str(animals),'=table(coef,A1,A2,B1,B2)']);

for age = 1:maxage+1

%% plot

bar([Data2(age).age(animals).animal.mean]);

title(['Animal #',mat2str(animals),', age ',mat2str(age-1)])

% xticklabels({'BB','A1B','BA2','A1A2'})

grid minor

set(gca,'FontSize',18)

%% talbel

Mean=[Data2(age).age(animals).animal.mean]';

Var=[Data2(age).age(animals).animal.var]';

Combination={'B1B2','A1B2','B1A2','A1A2'}';

Animal = [animals;animals;animals;animals];

Age = [age-1;age-1;age-1;age-1];

Talbel_for_mean_var(age*4-3:age*4,:)=table(Animal,Age,Combination,Mean,Var)

pause

if age == maxage+1

clearvars Cues Table_for_mean_var

end

end

end
