# Supplementary material for: Combining information from parental and personal experiences: Simple processes generate diverse outcomes
Source: PLoS One. 2021 Jul 13;16(7):e0250540. doi: 10.1371/journal.pone.0250540 (PMC8277055; doi:10.1371/journal.pone.0250540)
Supplement: S1 Fig — (DOCX) [file pone.0250540.s006.docx]

S1 Fig Parental Prior distributions
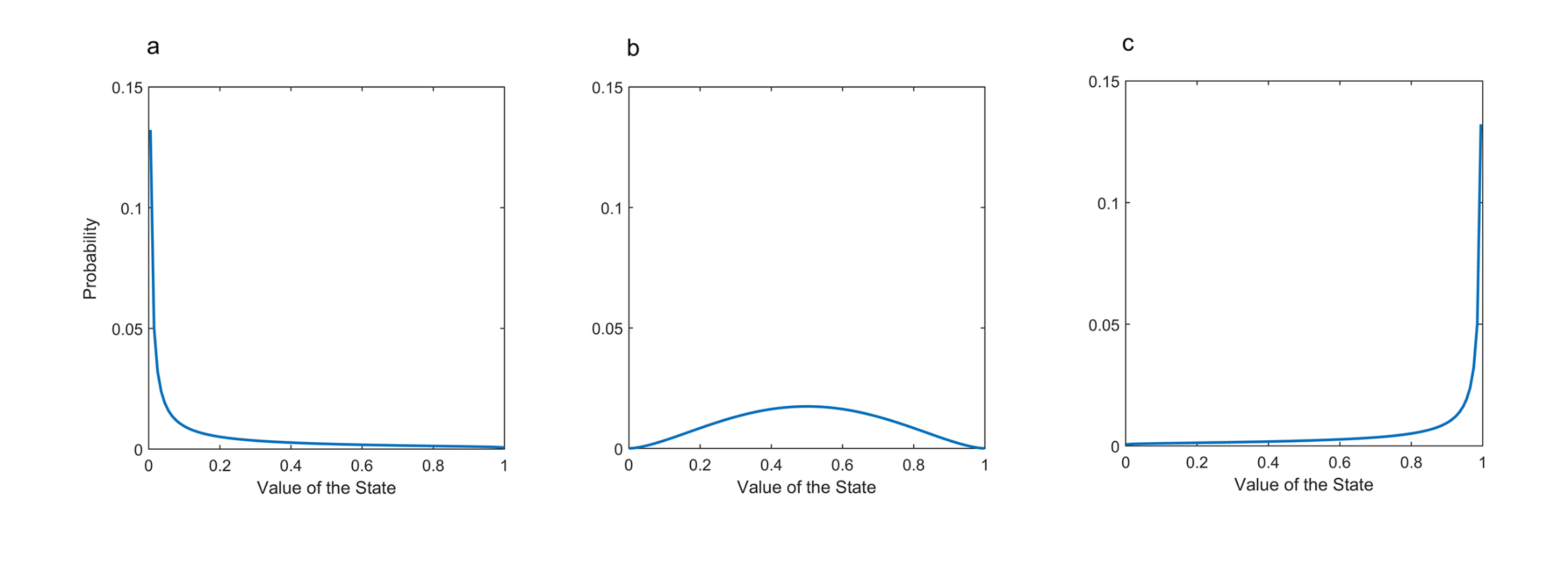


The parental Prior distributions used in this study. Each Prior represents the parent’s estimate of the probability of each of the 100 values of the state at the beginning of the study. All of these Prior distributions have the same variance (0.04).

a) Mean of the Prior = 0.1 b) Mean of the Prior = 0.5 c) Mean of the Prior = 0.9
