# Supplementary material for: Combining information from parental and personal experiences: Simple processes generate diverse outcomes
Source: PLoS One. 2021 Jul 13;16(7):e0250540. doi: 10.1371/journal.pone.0250540 (PMC8277055; doi:10.1371/journal.pone.0250540)
Supplement: S2 Fig — (DOCX) [file pone.0250540.s007.docx]

S2 Fig Cumulative Likelihood Functions


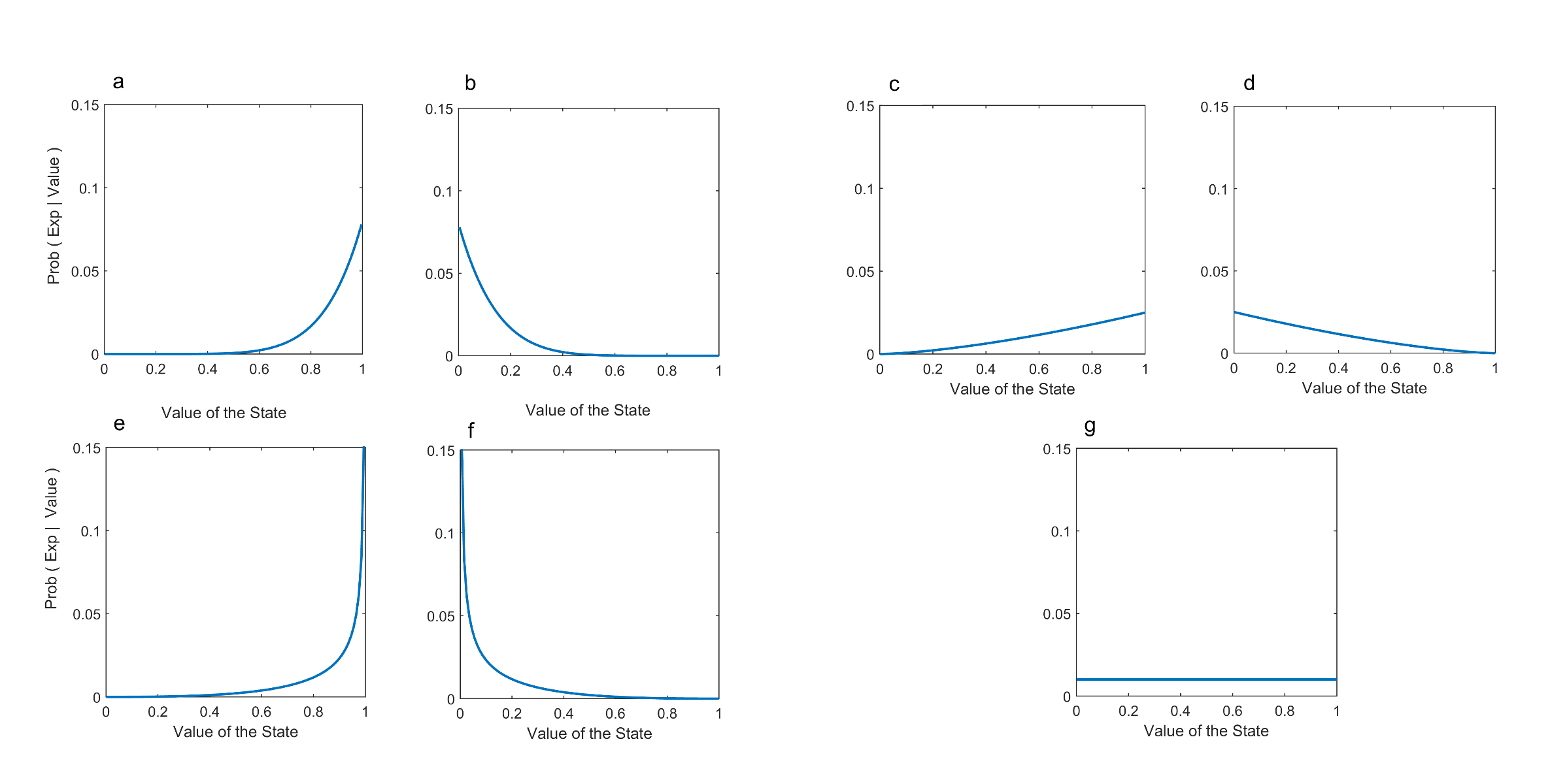


< See legend on the next page>

For a particular Experience (e.g., exposure to a specific concentration of kairomones from a predator for a specified period of time), the cumulative likelihood function indicates the conditional probability that an individual will have that Experience, given each of the 100 possible values of a state of the environment (e.g., predator density). The shape of each likelihood function conforms to a beta distribution specified by two parameters, α and β. The cumulative likelihood functions used in this article have the shapes indicated by beta distributions specified by the following parameter values: a) α = 8, β = 1; b) α = 1, β = 8; c) α = 2.5, β = 1; d) α = 1, β = 2.5; e) α = 3.5, β = .44; f) α = .44, β = 3.5 and g) α = 1 β = 1

Likelihood functions a) and b) are mirror-images of one another: a) indicates that the Experience is more likely to occur when values of the state are high than when they are intermediate or low; b) indicates the reverse.

Likelihood functions a) and e) both indicate that the Experience is more likely to occur when the values of the state are high than when they are intermediate or low, and both likelihood functions have the same mean value. However, the variance of the likelihood function for a) is lower than the variance of the likelihood function for e). This indicates that a) provides a more reliable estimate than does e) of the conditional probability of the Experience, given each of the possible values of the state.

The likelihood function illustrated in g) is the uniform distribution. This likelihood function indicates that the Experience is equally likely to occur given every possible value of the state. As a result, exposure to this likelihood function has no effect on the subject’s estimates of the true value of the state.
