# Supplementary material for: Combining information from parental and personal experiences: Simple processes generate diverse outcomes
Source: PLoS One. 2021 Jul 13;16(7):e0250540. doi: 10.1371/journal.pone.0250540 (PMC8277055; doi:10.1371/journal.pone.0250540)
Supplement: Attachment — Submitted filename: Review of Stamps and Bell for PLoS One.pdf [file pone.0250540.s008.pdf]

Review of "How basic components of information-updating interact to encourage variation in the results of empirical studies of within and transgenerational plasticity"

Overall comments

This paper continues an outstandling line of work [references 19, 21-25] concerning information, how it is updated in light of experience, and the implications for understanding behavioral and developmental plasticity. Although I have some quibbles about the writing (to be listed below) that can be fixed by minor reorganization, the paper is very well written and the interpretation of the modeling results explained in a clear way. This will be a very important contribution for bringing new conceptual frameworks to empiricists studying plasticity.

In addition, I believe that a new Supplementary Material is required, explaining the methods in more detail. I make suggestions about this at the end of this review.

Specific comments

lines 19, 35-36: I suggest that you write "trans and within generational plasticity" so that the acronym TWP makes sense.

line 45: There are people who will get bent out of shape when you say that Bayesian methods are the best, so you could better write this as "..Bayesian methods are a consistent and natural way to combine..."

line 63: will most readers know what naive priors are? It would be good to have a note indicating that this is described in the SM glossary (to which it needs to be added).

line 82: Exactly what empirical results are of interest here? If they are in any of your papers cited, then please indicate which ones. If not, think about adding a table concerning the empirical work that you are wanting to explain.

lines 90-92 and throughout: As far as I can tell, this is the first time that the words devalued and degraded are used. Although they are clear in an intuitive, colloquial sense, it is not clear what they mean in the information processing model. Please explain

It might be good to have a picture in the main text showing how the Bayesian updating works, and through that what devalued and degraded mean.

lines 113, 127, 134: These begin "Second", "In addition", "Second" and I lost the flow. Please fix that.

lines 149-153: It took eight pages to get to an explanation of what you are doing, but it will

be hard for readers to stick with it. I suggest that you move this up around line 103 and then let the following writing show why this is worth doing.

lines 156-173 and throughout. Why does prior have an upper case P? You also need to let the reader know what random variable this prior is characterizing. Please think about something other than saying “state of the environment” but rather give an example or two.

lines 191-194: How does gamete production get into here? I thought that the point was to focus on information states rather than detailed development.

lines 199-206: I cannot figure out what is going on here. In lines 203-204 do you mean probability of exposure to a predator or not?

lines 212-216: This is a nice overview of what you did and should come sooner.

lines 238-239. Now you are talking about variance of the likelihood, by which I presume you mean the entire beta density. It would be very good for the reader to thus know how the mean and variance of a beta density depends on its parameters, without having to go back to Stamps and Krishnan (2014a). All of these issues can be fixed if you have a SM figure that shows the three beta densities that you use.

line 299 following. It is not clear to me if these are captions for figures or discussion of the figures. But most importantly – what is unexpected in these results?

lines 448-452. You have already noted that one of the properties of Bayesian updating is that unless information is forgotten, the posterior usually is tighter (smaller variance) than the prior, so it is almost an *a priori* prediction that the lifetime experience of the parent provided to the offspring will have a stronger impact than early lifetime experiences of the offspring. Am I missing something?

lines 453-464. You began the paper saying that it you were focussing on information without the developmental state, so I suggest you let readers fill in this paragraph in their own minds. This also makes a smoother transition to the paragraph beginning on line 465

lines 496-510. I am not sure that you need this paragraph for the current manuscript, since you do not treat this question. If you decide to keep it, fine, but in line 510 the word data is plural so the sentence should read “Unfortunately, at present these data are still sparse [42-44].”

lines 511-532: Much of this is about degradation of information from the parents, but I still do not understand how that is treated in the model or what you mean by ‘noise’ (or why it is in quotation marks).

lines 533-540: This is the first time that the word inductive is used in the paper and it is not in your little glossary. I cannot figure out what this paragraph is about since I do not know what inductive cues or experiences are.

lines 552-587: This is all about development and I wonder if you need it. If you are setting

up future work in which this paper is linked to models of development, okay. But otherwise perhaps not.

line 683. This citation needs a date.

line 735, 739. Species name in italics

Figures: In all the figures, the prior is not 0.1, 0.5, or 0.9. Rather the mean of the prior takes those values.

List of terms

Why is the **estimate** only the mean of the prior or posterior distribution? The estimate is the entire distribution, you are choosing to use the mean – which is fine since you do not have any development in here. When development is a nonlinear function of the state, then we expect the entire distribution to play a role in development, not just its mean.

A New SM Describing the Model

Although the code is available, it is often very difficult to read other people's code, especially if one is trying to grasp the logic of the model rather than the details.

To help readers who want to replicate your work or build on it, I suggest that you provide either or both of

- A flowchart showing how the model works.
- Pseudo-code, which can refer to commonly used distributions such as the beta (e.g. a statistics book or an R library) but which gives equations for the unusual ones in your work.

Summary

This has the potential to be a really important and highly cited paper, given the currrent interest in transgenerational plasticity. I look forward to seeing a revision that has the SM with a description of the model.

Marc Mangel
